# Supplementary material for: A High-Quality Reference Genome Assembly of the Saltwater Crocodile, Crocodylus porosus, Reveals Patterns of Selection in Crocodylidae
Source: Genome Biol Evol. 2019 Dec 10;12(1):3635–46. doi: 10.1093/gbe/evz269 (PMC6946029; doi:10.1093/gbe/evz269)
Supplement: evz269_Supplementary_Data [file evz269_supplementary_data.zip › Supplementary_File_S5.docx]

**5’UTR**

**Exon**


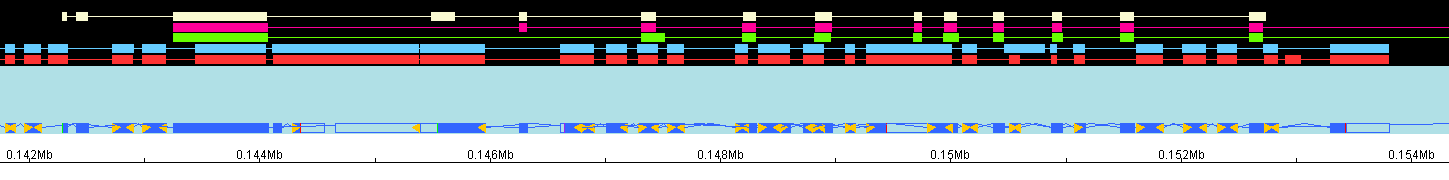


**Intron**

**Figure S2. A single gene prediction as predicted with the help of EST and protein evidence in presence of ab-initio gene predictor SNAP through the MAKER2 pipeline.** Protein and RNA transcript “evidence” from previously released *C. porosus* genome assembly was used to train SNAP and the pipeline in general. Figure was generated in the Apollo genome browser.

**3’UTR**

**Intron**

**Intron**

**Exon**

**Exon**

**Exon**

**3’UTR**

**Figure generated in Apollo genome browser**


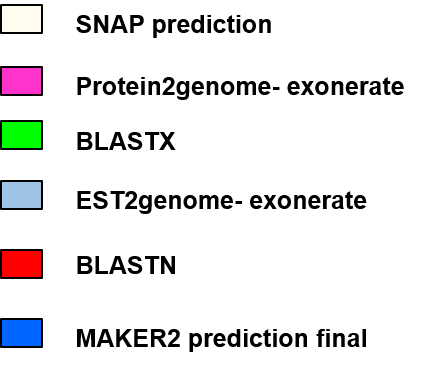


.
