# Supplementary material for: A High-Quality Reference Genome Assembly of the Saltwater Crocodile, Crocodylus porosus, Reveals Patterns of Selection in Crocodylidae
Source: Genome Biol Evol. 2019 Dec 10;12(1):3635–46. doi: 10.1093/gbe/evz269 (PMC6946029; doi:10.1093/gbe/evz269)
Supplement: evz269_Supplementary_Data [file evz269_supplementary_data.zip › Supplementary_Methods_GBE_cPorosus_Genome.docx]

***SUPPLEMENTARY METHODS***

***Additional details and options from MAKER run***

The first MAKER2 run was performed only in presence of transcript and protein evidences from the previous saltwater crocodile genome annotation. The est2genome and protein2genome and AED option were all set to 1. This step was run without any ab-initio gene predictors. MAKER2 primarily accomplishes this using BLAST (ver. 2.6.0) (Altschul et al. 1990) and Exonerate (ver. 2.2.0) (Slater and Birney 2005). However, BLAST fails to take into account splice sites and junctions. Exonerate, being a splice-site aware aligner, precisely addresses this issue by using its own algorithm to realign and polish the sequences after filtering and clustering. “Polish” refers to realigning BLAST alignments a second time with information of splice donor and acceptors, thus improving the precision at exon boundaries. On the other hand, “filtering” and “clustering” are two independent but closely related procedures where filtering identifies and removes marginal predictions and sequence alignments based on scores and percent identities. Clustering, refers to collecting groups of different computation data, which supports identical gene and transcript as well as identifying redundant data.

In the second round once SNAP was trained using the GFF file generated above, both the est2genome and protein2genome options were set to 0. However the evidence FASTA files were still provided. The AED score was set to 0.3 to minimize false positives. The AED is a distance metric score, developed by the Sequence Ontology Project (Eilbeck et al. 2009) with values ranging from 1 through 0. Gene models with scores closer to 0 signifies better alignment of predicted gene models with the transcript evidence in the MAKER2 pipeline. The option “min_contig” value was set to 10,000, as true eukaryotic genes are rarely observed with a size < 10kb. This resulted in skipping contigs less than 10kb in size during analysis.

***Training the ab-initio gene predictor SNAP***

To train SNAP, first the GFF file previously generated by the standalone run of MAKER2 was converted into ZFF format. ZFF is a format tailor-made for the SNAP tool and the content of the ZFF files bear close resemblance to GFF and FASTA files (Korf 2004).

***Microsatellite Identification***

For microsatellite identification, of the 282 loci- 34 did not map to the genome, 155 mapped uniquely to a single location in the genome and 93 mapped to two or more places in the genome. Of the 34 loci that did not map to the genome, 22 loci (KX055916.1 -KX055937.1) had the same sequence and can thus be considered as a single microsatellite locus. This locus was mapped to the genome with Cj16 primers (Isberg et al. 2004) using the *in-silico* primer mapping algorithm in Geneious v 10.0.9.

Among loci with two or more positions within the same contig, the distance was <900 bp about 97% of the time. On closer examination in IGV, we observed that such loci had masked repeat sequences interspersed between two mapping positions. Hence, the first mapping position was selected for such loci and the other position removed from further analysis. The remaining 3% of loci with distances >900 bp was not included in the analysis.

***InterProScan5 options***

The InterProScan5 program was run on the BLAST annotated protein FASTA file with the “–goterms iprlookup” option enabled, which allowed searching of corresponding Gene Ontology options through InterProScan. The output .tsv file of InterProScan5 was used with the “ipr_update_gff” script to add the InterProScan information to the functional annotated GFF file.

***Gene branchlength analysis***

For the reconstructed crocodilian ancestor genome assembly, MAKER2 pipeline using the exact similar procedure as described in the main manuscript was run and genes were predicted. SNAP was used again as the ab-initio gene predictor. ProteinOrtho was run using reconstructed ancestral crocodilian assembly as well as chicken (*Gallus gallus*) as the outlier groups for comparison. BLAST was used to make protein databases from the protein FASTA sequences for all the concerned species. “makeblastdb” function was used with “dbtype = prot”. These databases were needed to run ProteinOrtho. For MAFFT, the options “--maxiterate 1000” and “--localpair” were used. After PAML was run, a ratio of the branch length of the crocodile to the alligator was evaluated. The file was sorted based on highest branch length ratio value. The top 5% of these reflected genes potentially evolving faster in the crocodile, while the bottom 5% represented genes evolving rapidly in the alligator.

**Statistical test for adaptive evolution of codons**

The statistical test for adaptive evolution of codons was performed using both the American alligator and saltwater crocodile as well as two outgroups- the ancestral crocodylian assembly (reconstructed previously using Progressive cactus in Green et al. 2014) and chicken. Pal2nal (Suyama et al. 2006) was run with the options –output paml and –nogap. These options were essential for generating input files for CODEML in the next step. Additional input files provided included the CDS and aligned protein sequences for all the 2283 and 2357 single-copy orthologous genes using the reconstructed ancestral crocodylian genome and chicken outgroups respectively. The following site-selection models were tested for the crocodylian species- M0, M1, M2, M7 and M8 (Yang et al. 2000; Anisimova et al. 2001; Swanson et al. 2001; Yang and Nielsen 2002). Using the chi-square distribution table, only if we found statistical significance for log-likelihood values of the M0-M1 pair (M0 being nested within M1), we proceeded to validate the M1-M2 and M7-M8 pairs. The M1 and M7 models test for neutral to purifying selection while the M2 and M8 models test for positive selection. With M0-M1 validated, only if there was a statistical significance over the log-likelihood values of M7-M8 pair, we considered the orthologous gene to be under potential positive selection. The following optional values were set in the control file for the CODEML analysis. runmode = 0 (user newick tree was supplemented); clock = 0; seqtype = 1; CodonFreq = 2; model = 0; NSsites = 0 1 2 7 8; fix_omega = 0; omega = 0.4 and icode = 0.

**GO-term enrichment for genes and potential gene networking pathways in the saltwater crocodile**

KOBAS 3.0 (Wu et al. 2006; Xie et al. 2011) was used to analyze GO term enrichment for orthologous genes and analyzing the pathways in which these GO enriched genes were involved. KOBAS 3.0 server (http://kobas.cbi.pku.edu.cn/index.php) was run online and input files included orthologous FASTA peptide sequences from the saltwater crocodile analyzed through the codon site model of CODEML. Human was chosen as reference in both KOBAS 3.0 and STRING (https://string-db.org/) (Szklarczyk et al. 2015) analyses due to best annotation of reference genes across all datasets. This helps us most in analyzing liked genes and the pathways they are involved (enriched) in. KOBAS 3.0 includes multiple databases for its analysis and includes databases like KEGG PATHWAY, Reactome, Biocyc and Panther. The P-value of significance generated upon running its algorithms is not sufficient. The P-value needs to be corrected of type I error since a large number of pathways across multiple databases are cross-referenced and multiple hypothesis tests are performed. This greatly increases chances of type I error. Hence FDR correction is applied and the corrected P-value of significance (P ≤ 0.05) is chosen as a cut-off.

For STRING, the option “multiple sequences” was chosen to analyze all orthologous protein sequences.

**References**

Altschul SF, Gish W, Miller W, Myers EW, Lipman DJ 1990. Basic local alignment search tool. Journal of molecular biology 215: 403-410.

Anisimova M, Bielawski JP, Yang Z 2001. Accuracy and power of the likelihood ratio test in detecting adaptive molecular evolution. Molecular biology and evolution 18: 1585-1592.

Eilbeck K, et al. 2005. The Sequence Ontology: a tool for the unification of genome annotations. Genome biology 6: R44.

Green RE, et al. 2014. Three crocodilian genomes reveal ancestral patterns of evolution among archosaurs. Science 346: 1254449.

Isberg S, Chen Y, Barker S, Moran C 2004. Analysis of microsatellites and parentage testing in saltwater crocodiles. Journal of Heredity 95: 445-449.

Korf I 2013. SNAP: Semi-HMM-based Nucleic Acid Parser. Ian Korf homepage: http://homepage. mac. com/iankorf.

Slater GSC, Birney E 2005. Automated generation of heuristics for biological sequence comparison. BMC bioinformatics 6: 31.

Suyama M, Torrents D, Bork P 2006. PAL2NAL: robust conversion of protein sequence alignments into the corresponding codon alignments. Nucleic acids research 34: W609-W612.

Swanson WJ, Yang Z, Wolfner MF, Aquadro CF 2001. Positive Darwinian selection drives the evolution of several female reproductive proteins in mammals. Proceedings of the National Academy of Sciences 98: 2509-2514.

Szklarczyk D, et al. 2015. STRING v10: protein-protein interaction networks, integrated over the tree of life. Nucleic Acids Res 43: D447-452. doi: 10.1093/nar/gku1003

Wu J, Mao X, Cai T, Luo J, Wei L 2006. KOBAS server: a web-based platform for automated annotation and pathway identification. Nucleic Acids Res 34: W720-724. doi: 10.1093/nar/gkl167

Xie C, et al. 2011. KOBAS 2.0: a web server for annotation and identification of enriched pathways and diseases. Nucleic Acids Res 39: W316-322. doi: 10.1093/nar/gkr483

Yang Z, Bielawski JP 2000. Statistical methods for detecting molecular adaptation. Trends in ecology & evolution 15: 496-503.

Yang Z, Nielsen R 2002. Codon-substitution models for detecting molecular adaptation at individual sites along specific lineages. Molecular biology and evolution 19: 908-917.
